# Dragging Human Mesenchymal Stem Cells with the Aid of Supramolecular Assemblies of Single-Walled Carbon Nanotubes, Molecular Magnets, and Peptides in a Magnetic Field

**DOI:** 10.1155/2015/143504

**Published:** 2015-01-26

**Authors:** Ana Cláudia C. de Paula, Gustavo A. M. Sáfar, Alfredo M. Góes, Marcelo P. Bemquerer, Marcos A. Ribeiro, Humberto O. Stumpf

**Affiliations:** ^1^Departamento de Bioquímica e Imunologia, Instituto de Ciências Biológicas, Universidade Federal de Minas Gerais, 31270-901 Belo Horizonte, MG, Brazil; ^2^Departamento de Química, Universidade Federal de Minas Gerais, 31270-901 Belo Horizonte, MG, Brazil; ^3^EMBRAPA Recursos Genéticos e Biotecnologia, 70770-917 Brasília, DF, Brazil

## Abstract

Human adipose-derived stem cells (hASCs) are an attractive cell source for therapeutic applicability in diverse fields for the repair and regeneration of damaged or malfunctioning tissues and organs. There is a growing number of cell therapies using stem cells due to their characteristics of modulation of immune system and reduction of acute rejection. So a challenge in stem cells therapy is the delivery of cells to the organ of interest, a specific site. The aim of this paper was to investigate the effects of a supramolecular assembly composed of single-walled carbon nanotubes (SWCNT), molecular magnets (lawsone-Co-phenanthroline), and a synthetic peptide (FWYANHYWFHNAFWYANHYWFHNA) in the hASCs cultures. The hASCs were isolated, characterized, expanded, and cultured with the SWCNT supramolecular assembly (SWCNT-MA). The assembly developed did not impair the cell characteristics, viability, or proliferation. During growth, the cells were strongly attached to the assembly and they could be dragged by an applied magnetic field of less than 0.3 T. These assemblies were narrower than their related allotropic forms, that is, multiwalled carbon nanotubes, and they could therefore be used to guide cells through thin blood capillaries within the human body. This strategy seems to be useful as noninvasive and nontoxic stem cells delivery/guidance and tracking during cell therapy.

## 1. Introduction

The regenerative medicine with mesenchymal stem cells (MSCs) is becoming more frequent with the recent studies highlighting the potential of MSCs applicability in diverse fields for the repair and the regeneration of damage tissues [1]. Therefore, the development of methods to monitor stem cells delivery on target organ of interest and track cell retention is required [2]. Magnetic delivery of stem cells using nanoparticles has the potential to become a powerful tool in regenerative medicine. However, the appropriate magnetic nanoparticle must be carefully chosen depending on the task.

A number of studies have shown that iron-based nanoparticles, particularly those made from iron oxide, can cause undesired effects and interfere with normal cellular processes [3, 4]. Because these negative effects of Fe^3+^ are still unknown, iron-based nanoparticles appear to be a poor choice and should only be used if tumour destruction is the goal. Therefore, they should be avoided in the case of medicine regenerative.

Recently, single-walled carbon nanotubes (SWCNT) and multiwalled carbon nanotubes (MWCNT) have been successfully used as stem cell culture substrates without disabling the cell's ability to differentiate [5]. Studies showed that SWCNT did not activate immune proteins [6] and modified surface of the nanotubes could minimize immune response [7]. A recent study demonstrated the first use of nanotubes as magnetic stem cell carriers using MWCNT [8].

The advantages of using SWCNT, compared to MWCNT, is the characteristic Raman-active radial breathing mode and the huge Raman cross-section related to them, which make them traceable [9, 10]. And, as the SWCNT are narrower, they can be preferentially used to guide stem cells in thin capillaries within the body. Another difference is that SWCNT may carry metal alloys as MWCNT, but they are much smaller than the MWCNT cores and postsynthesis treatments can often be performed to eliminate them. Nevertheless, nanoparticle-free SWCNT are superparamagnetic, which ensures that they can be dragged by a magnetic field gradient [11–15], meaning that a pure SWCNT sample is attracted by a small NdFeB magnet in its proximity, due to the gradient field.

In this study, we propose a SWCNT supramolecular assemblies (SWCNT-MA) consisting of SWCNT, molecular magnets, and a peptide for anchor and move human adipose-derived stem cells (hASCs) by an applied magnetic field. In the attempt to develop a SWCNT-MA with minimal metallic nanoparticle content to drag stem cells through the body.

## 2. Material and Methods

SWeNT SWCNT (CoMoCAT) were purchased from SouthWest NanoTechnologies Inc. for this study. These SWCNT showed no electron paramagnetic resonance (EPR) signal at room temperature. Energy dispersive spectroscopy (EDS) and thermogravimetric analysis (TGA) indicated a metallic nanoparticle content of less than 0.1% of weight as previously described by one of our group [13].

### 2.1. Synthesis of Lawsone-Co-Phenanthroline

First, 0.5 mmol of lawsone was dissolved in 20 mL of 2-propanol, and 0.5 mmol of triethylamine was added over a stirrer. To this solution, a separate solution of Co(ClO_4_)·6H_2_O in 10 mL of 2-propanol was added dropwise. Then, a solution of 0.5 mmol of phenanthroline in 2-propanol was added over a stirrer. The resultant solution was left to evaporate for 5 days; at each time suitable single crystals were collected.

### 2.2. Peptide Synthesis

The peptide was synthesized at the 0.15 mmol scale by using the Fmoc solid-phase methodology [16]. A Rink Amide-MBHA resin (0.52 nmol/g; Peptides International, Louisville) was used to obtain the amidated peptide. Couplings were conducted by carboxyl group activation with 1,3-diisopropylcarbodiimide/[ethyl 2-cyano-2-(hydroxyimino) acetate [17] or with [benzotriazol-1-yloxy(dimethylamino)methylidene]-dimethyl-azanium tetrafluoroborate (TBTU)/diisopropylethylamine (DIPEA) in N,N-dimethylformamide (DMF, 3.0 mL). Fmoc-amino acids and coupling reagents were used at 4 molar excess to the amino group. Deprotections were performed in 2 steps (15 minutes each) with 4-methylpiperidine/DMF (25/75, by volume). After the cleavage and deprotection steps, alternating washings were conducted with 2-propanol and with DMF, 4 times each, which were followed by the ninhydrin qualitative assay. Final deprotection and peptidyl-resin cleavage were conducted using a solvent mixture containing trifluoroacetic acid (TFA)/methylphenylsulfide/water/phenol/1,2-ethanedithiol/triisopropylsilane (81.5/5.0/5.0/5.0/2.5/1.0, by volume). Crude peptide was solubilised in aqueous acetonitrile (50% by volume) and then freeze-dried. The peptide was purified by reversed-phase chromatography at room temperature by using a Zorbax ODS column (9.4 × 250 mm) from Agilent (Santa Clara, CA). The gradient program was as follows: H_2_O/ACN/TFA (95/5/0.1; v : v : v) for 10 minutes, followed by a linear gradient to H_2_O/ACN/TFA (5/95/0.1; v : v : v) for 90 minutes with detection at 220 and 280 nm and a flow rate of 3.0 mL·min^−1^. Peptide identity was confirmed by MALDI mass spectrometry (Ultraflex III Bruker Daltonics, Bilerica, MA), with analysis in the positive mode using *α*-cyano-4-hydroxycinnamic acid as the matrix.

### 2.3. Synthesis of SWCNT-MA

First, 4.5 mg of lawsone-Co-phenanthroline (L18) and 3 mg of SWCNT were added to 5 mL of dimethylsulfoxide and sonicated for 20 minutes with a high-power tip sonicator. The mixture was then dried in a vacuum drier at room temperature. Then, 5.9 mg of the dry product (SWCNT/L18) was resuspended in 1.0 mL deionized water with sonication for 10 minutes. The SWCNT/L18 was then added to 10 mL of a water solution containing 3.7 mg of the FWYANHYWFHNAFWYANHYWFHNA peptide (determined by optical absorption at *λ* = 280 nm). The role of the peptide is to be a biocompatible surfactant for the nanotubes. The peptide sequence is composed of many aromatic amino acids, which can easily adhere on the nanotube wall, while the hydrophilic portions can solvate the SWCNT-MA on the aqueous fraction.

The whole mixture with a total concentration of 0.87 mg/mL was then sonicated for 2 minutes. The redundant peptide was not extracted from the SWCNT-MA suspension. The final SWCNT-MA suspension (with the redundant peptide) was stocked for administration to the culture cells.

### 2.4. Isolation and Culture of hASCs

Human adipose tissue was harvested from healthy patients who had abdominal reduction surgery. All the donors provided written informed consent. The approval number in the Ethics Committee in Research from Federal University of Minas Gerais is—ETIC 0023.0.203.000-11. The hASCs were obtained and cultured as described previously by Zuk et al. [18]. The cells were cultured in basal medium Dulbecco's modified Eagle's medium-high glucose (Sigma-Aldrich) supplemented with 5 mM sodium bicarbonate, penicillin (100 units/mL), streptomycin (0.1 mg/mL), amphotericin B (0.25 *μ*g/mL) (Sigma-Aldrich), gentamicin (60 mg/L, Schering-Plough), and 10% of fetal bovine serum (FBS) (Cripion Biotecnologia LTDA, Brazil) at 37°C in a 5% CO_2_ humidified atmosphere. And the hASCs were cultured under basal medium with SWCNT-MA suspension (diluted 1/5 or diluted 1/10 in basal medium) to perform the assays.

### 2.5. Flow Cytometry

Immunophenotypic analyses were performed with hASCs. In short terms, the cells were incubated with each monoclonal antibody for 30 minutes at 4°C. Then, the cells were washed twice with PBS. For some groups of markers (unconjugated antibodies), the cells were incubated with a secondary antibody for 30 minutes at 4°C and were protected from light. Finally, the cells were washed in PBS and fixed with 200 *μ*L of 1% formaldehyde. The hASCs were incubated with only Alexa Fluor 488 goat anti-mouse immunoglobulin G (IgG) (Invitrogen) to assess the background fluorescence of the secondary antibody as a control. The following unconjugated mouse monoclonal antibodies were used: integrin *β*1chain—CD29 (Santa Cruz Biotechnology), HCAM—CD44 (Santa Cruz Biotechnology), hematopoietic stem cell- (HSC-) associated marker—CD34 (Abcam), and pan-leukocyte marker CD45 (BD Biosciences), which were stained with Alexa Fluor 488 goat anti-mouse IgG as a secondary antibody. The following conjugated antibodies were used: ecto-5′ nucleotidase nucleotidase-CD73-phycoerythrin (BD Biosciences), human leukocyte antigens- (HLA-) ABC-fluorescein isothiocyanate (FITC) (Abcam), and HLA-DR-FITC (Abcam). The hASCs were analyzed by flow cytometry with FACSCalibur (Becton Dickinson Immunocytometry System) using CELLQuest acquisition software (BD Biosciences). A minimum of 20,000 events were acquired for each sample of cells analyzed for each surface marker. Cell marker expression was determined by comparison with control and the data were analyzed with FlowJo software (Tree Star, San Carlos, CA, USA).

### 2.6. Multilineage Differentiation of hASCs

hASCs were analyzed for their capacity to differentiate toward the adipogenic, osteogenic, and chondrogenic lineages. To induce differentiation, cells were cultured with specific induction media, as detailed in Table 1 for 21 days [18]. Differentiation was confirmed using the histological assays. Adipogenic differentiation was induced by culturing hASCs for 21 days in a 6-well plate in adipogenic medium (AM) and assessed using an Oil Red O (Thermo Scientific) stain as an indicator of intracellular lipid accumulation. Prior to staining, the cells were fixed for 60 minutes at room temperature in 10% formalin and washed with 60% 2-propanol. The cells were incubated in Oil Red O solution in 60% 2-propanol for 5 minutes. Excess stain was removed by washing with water. The cells were counterstained for 1 minute with hematoxylin. Osteogenic differentiation was induced by culturing hASCs for 21 days in a 6-well plate in osteogenic medium (OM) and the cultures were examined for extracellular matrix (ECM) calcification by von Kossa staining. For von Kossa staining, the cells were fixed with 70% ethanol for 24 hours at room temperature. The cells were rinsed with distilled water and then overlaid with a 5% silver nitrate solution and exposed to ultraviolet light for 60 minutes. The cells were rinsed with distilled water and 5% sodium thiosulfate for 5 minutes. Finally, the cells were counterstained with eosin. Chondrogenic differentiation was induced culturing the hASCs in a three-dimensional pellet. Briefly, 5 × 10^5^ cells were seeded into 15 mL polypropylene conical tube and centrifuged at 800 g for 5 minutes. Chondrogenic medium (CM) was gently overlaid so as not to detach the cell pellets, and cultures were maintained in CM for 21 days. Chondrogenesis was confirmed using the histologic stain Alcian Blue 8GX (1% in acetic acid, pH 2,5) performed in sections of pellets showing specific staining of sulfated proteoglycans and glycosaminoglycans, present in cartilaginous matrices.

### 2.7. Cellular Viability

Cell viability and proliferation were assessed by performing an MTT assay, as previously described by Mosmann [19]. The hASCs were plated in a density of 5 × 10^4^ cel/well in a 24-well plate and cultured for 2 or 6 days in 1 mL of basal medium with SWCNT-MA suspension, which was diluted 1/5 or diluted 1/10 in basal medium. Each time, the medium was removed and 170 *μ*L of MTT (5 mg/mL) solution and new basal medium were added to each well. Two hours later, the formazan crystals were dissolved using sodium dodecyl sulphate- (SDS-) 10% HCl. After 18 hours of incubation at 37°C in a 5% CO_2_ humidified atmosphere, 100 *μ*L of solution was transferred to a 96-well plate and the optical density (OD) was measured at 595 nm.

Moreover, for the assessment of the cell morphology and viability, hASCs cultured for 10 days in basal medium containing SWCNT-MA (diluted 1/5 or diluted 1/10 in basal medium) were seeded at a density of 2 × 10^4^ cells/cm^2^ onto tissue culture polystyrene coverslips (2.2 cm × 2.2 cm) and were incubated for 20 minutes in PBS containing 0.1 mM calcein acetoxymethyl ester (Calcein-AM). Calcein-AM is a nonfluorescent, cell-permeant compound that is hydrolyzed by intracellular esterases into the fluorescent anion calcein. The Calcein-AM stained cells were visualized through confocal microscopy (Zeiss LSM 5 Live). The cellular viability was evaluated for cells cultured with the basal medium and the basal medium with 1/5 and 1/10 SWCNT-MA suspension.

### 2.8. Alkaline Phosphatase Activity

The alkaline phosphatase (AP) activity was evaluated by a BCIP-NBT kit assay as described by the manufacturer (Invitrogen). The hASCs were plated in a density of 5 × 10^4^ cells/well in a 24-well plate and cultured with 1 mL of basal medium with SWCNT-MA suspension, which was diluted 1/5 or diluted 1/10. At days 2 and 6 of culture, the supernatant of each well was removed and the cell layers were washed twice with PBS. Then, 200 *μ*L of BCIP-NBT solution was added to each well. Two hours later, the cells were observed by optical microscopy, and any visible purple precipitates were dissolved using SDS-10% HCl. After 18 hours of incubation at 37°C in a 5% CO_2_ humidified atmosphere, 100 *μ*L of solution was transferred to a 96-well plate, and the OD was measured at 595 nm.

### 2.9. Raman Measurements

hASCs were cultivated with no SWCNT (control aggregates) and with the addition of 50 *μ*L or 100 *μ*L of SWCNT-MA in the culture. Raman spectra were obtained upon observing cell aggregation and in the presence of the magnetic field, at 24 and 48 hours after cultivation.

Raman spectra were measured in the RBM spectral region of SWCNT, using a Senterra confocal Raman microspectrometer (Bruker) with an excitation of *λ* = 785 nm. Video sequences were filmed using a web camera facing the monitor of the computer that controls the spectrometer.

The magnetic field was generated by a 2.5 cm × 2.5 cm × 1 cm NdFeB permanent magnet with a surface field of 0.4 T, which measured 0.3 T at a distance of 0.5 cm.

## 3. Results and Discussion

**Figure 1:**
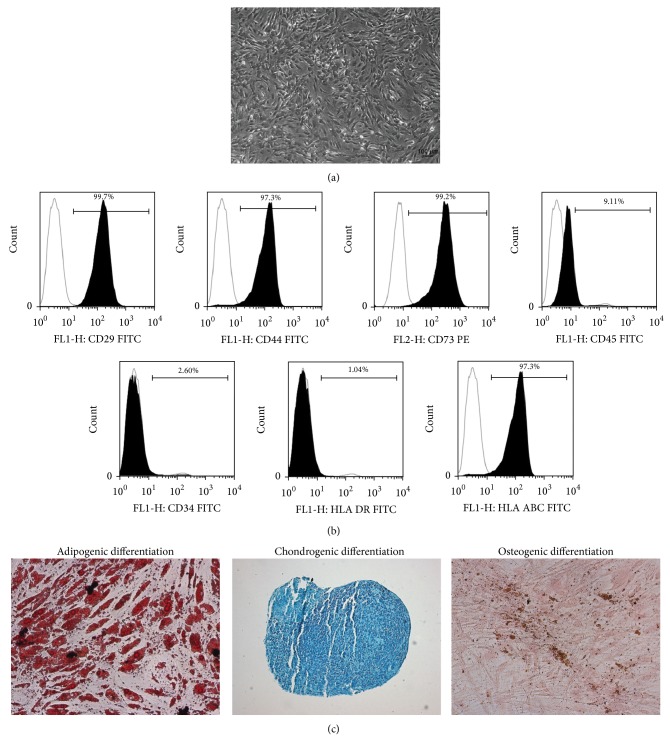
Human adipose-derived stem cells (hASCs) characterization. (a) Light micrograph of hASCs cultured in basal medium. (b) Cell phenotype analyzed by flow cytometry, expression of the selected mesenchymal stem cell, and hematopoietic and human leukocyte antigens markers are depicted with representative histograms. The white peak indicates the isotype-matched monoclonal antibody control. The black peak indicates positively stained cells. The cell populations expressed CD 29, CD 44, CD 73, and HLA-ABC and did not express CD 45, CD 34, and HLA-DR. (c) Multipotentiality of hASCs. Oil red O staining confirming adipogenesis, Alcian blue staining verifying chondrogenesis and von Kossa staining confirming osteogenesis.

The hASCs isolated from lipoaspirates presented fibroblast-like morphology, plastic-adherent (Figure 1(a)) and expressed CD29 (99.7% ± 0.3%), CD44 (97.3% ± 1.2%), CD73 (99.2 ± 0.5%), and HLA-ABC (99.7% ± 0.02%), and lacked expression of CD45 (9.1% ± 0.0%), CD34 (2.6% ± 0.6%), and HLA-DR (1.04% ± 0.0%) surface molecules (Figure 1(b)). Moreover the hASCs were able to differentiate to adipogenic, chondrogenic, and osteogenic lineages* in vitro* (Figure 1(c)). These characteristics are in accordance with criteria proposed by the Mesenchymal and Tissue Stem Cell Committee of the International Society for Cellular Therapy to define human MSCs [20].

**Figure 2:**
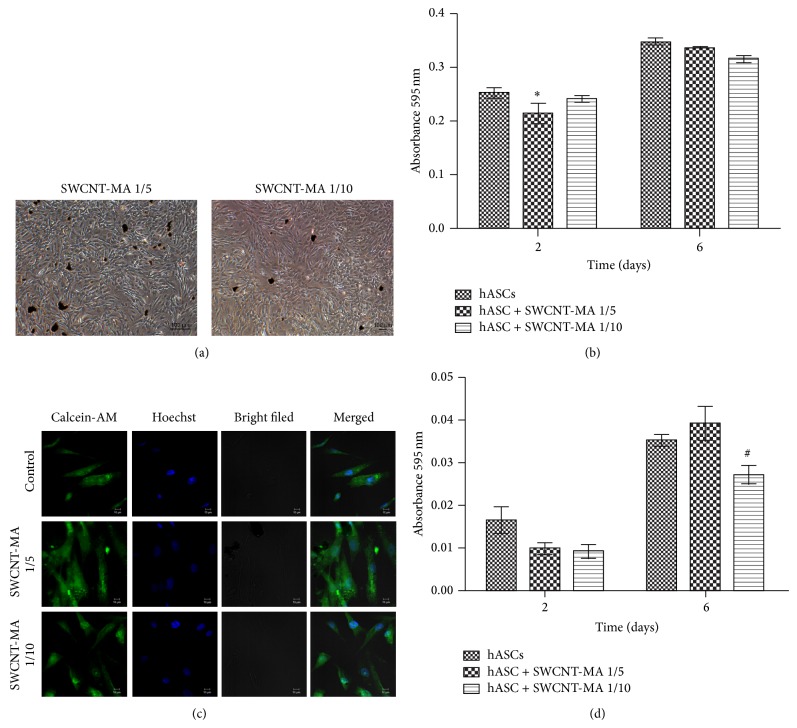
Human adipose-derived stem cells (hASCs) characteristics during culture with SWCNT-MA. (a) Light micrograph of hASCs cultured in basal medium SWCNT-MA suspension, which was diluted 1/5 or diluted 1/10. (b) MTT of the hASCs cultivated with SWCNT-MA suspension. (c) Cell viability after 10 days of cell culture in basal medium with SWCNT-MA suspension, which was diluted 1/5 or diluted 1/10 as analyzed by Calcein-AM staining. The results showing no toxicity of SWCNT-MA to hASCs cultures. (d) AP activity assay of the hASCs cultivated with SWCNT-MA suspension. 2-way ANOVA, Bonferroni posttest (*n* = 3). The results represent the mean ± SEM. ^*^ *P* < 0.05: hASCs × hASCs + SWCNT-MA 1/5 at time 2 days. ^#^ *P* < 0.05: hASCs + SWCNT-MA 1/5 × hASCs + SWCNT-MA 1/10 at time 6 days.

The presence of SWCNT-MA in cultures of hASCs resulted in aggregates of these cells forming black structures in some points as shown by Figure 2(a). The hASCs maintained their morphological characteristics, showing fibroblast-like morphology and adherent to plastic surface under the presence of SWCNT-MA suspension, diluted 1/5 or diluted 1/10 in basal medium (Figure 2(a)).

After 2 days of the seeding of the cells for the MTT assay, the absorbance measured for the SWCNT-MA 1/5 group was significantly reduced which can be due to an inefficient recovery of the procedure of seeding of the cells for this experiment compared with the other groups. The cells cultured in the presence of the SWCNT-MA suspension were able to metabolize the MTT producing formazan (Figure 2(b)), which demonstrated that the cells were metabolically active and the measurement of formazan produced showed that regardless of concentration of SWCNT-MA in the culture for 6 days, the cells were still viable and proliferated, which are in agreement with other study [8].

Moreover, cell viability assay employing Calcein-AM staining was performed to ensure the viability and assess the morphology of hASCs cultured with basal medium with SWCNT-MA suspension. These results showed that the cells cultured with SWCNT-MA were well adhered on the coverslips with fibroblastoid morphology that are characteristic of hASCs cultured with basal medium only and were viable as observed by the fluorescent anion calcein (Figure 2(c)). Alkaline phosphatase is an embryonic stem cell marker that has been previously reported to be expressed in human MSCs. Our results with hASCs cultured with SWCNT-MA suspension showed the typical pattern of AP activity, with the increase in alkaline phosphatase absorbance reflecting the higher cell number in culture after 6 days (Figure 2(d)). Direct observation of the cultures verified the maintenance of MSCs characteristics. Thereby, the SWCNT-MA demonstrated nontoxicity to hASCs. There is no direct evidence of SWCNT-MA internalization in the cells. However, the cells did not show evidence of toxicity. If there was some internalization, the SWCNT-MA intracellular concentration is below toxic levels.

**Figure 3:**
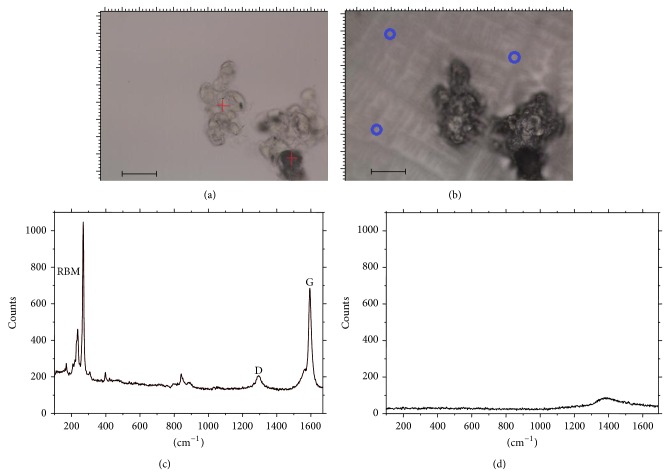
Microscopy image of the stem cells after addition of SWCNT-MA and growth along with their Raman spectra. (a) The red crosses indicate where the RBM Raman mode was found. Bar = 10 *μ*m. (b) Image of the same region after cells were dried. (c) Raman spectra of the red-cross positions in (a). The other characteristic Raman bands, which are related to SWCNT, are also indicated (G, D). (d) The spectra obtained from the blue dots (dried buffer solution). Only a small luminescence appears around 1400 cm^−1^.

Figure 3 shows a microscopic view of the cell aggregates, along with their Raman spectra. The red crosses indicate the points where Raman spectra were obtained, showing the RBM characteristic frequency of SWCNT. Spectra were also obtained from the buffer solution (locations indicated by the blue circles), and no traceable SWCNT was found in the buffer solution after 24 hours (Figure 3). The measurements were also obtained in dry aliquots of the buffer solution, where cells were absent, in order to enhance the Raman signal, if there was any signal. The RBM mode was not found in the buffer solution, indicating that SWCNT-MA had indeed adhered to the cell aggregates. Between 10 and 20 cell aggregates were tested for RBM mode signals in each well in order to improve the statistics. Few cells (<5%) were found unattached to any SWCNT. The RBM mode was undetectable at some aggregates which responded to magnetic field. However, the majority (90%) of the aggregates which responded did show the RBM mode signal. Video sequences were recorded and are available online (see Video 1 in Supplementary Material available online at http://dx.doi.org/10.1155/2015/143504).

The cultures with 100 *μ*L of SWCNT-MA suspension seemed to saturate the cell aggregate surface, which can improve the magnetic force used to drag them but may also damage the interface for cell differentiation and cause adhesion to live tissue. At the same time, the cultures with only 50 *μ*L of SWCNT-MA, which still showed an exposed aggregate surface, could be dragged by the magnetic field. The best incubation time appeared to be 24 hours, when the aggregates were composed of few cells and could be easily dragged by the magnetic field.

**Figure 4:**
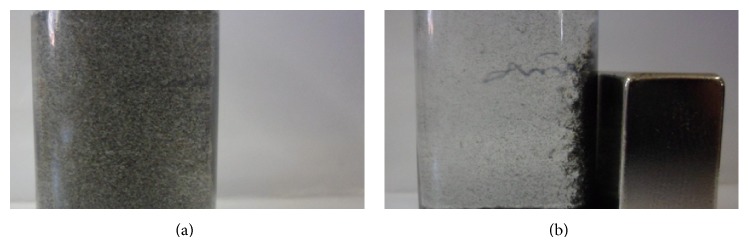
SWCNT-MA suspension orientation. (a) A suspension of SWCNT-MA in a glass vial. (b) The same suspension with an applied magnet field (originated by the NdFeB magnet at right).

The SWCNT-MA suspension with and without an applied magnetic field is shown in Figure 4. The aggregation on the vial sidewall where the NdFeB magnet was left shows the ferromagnetism of the SWCNT-MA. To show the practical application of the method, we can make a comparison. For instance, a magnetic field of 3 T is currently used for patients undergoing MRI scans, without disturbing physiological functions [21, 22]. In our experiments, the field never surpassed 0.3 T, which is 10 times lower than that commonly used for MRI. In fact, the magnet used in the study has a 10-fold weaker field than a 3-Tesla magnet used in common MRI equipment. Hence, around a 3-fold larger distance can be expected to have a similar effect on the SWCNT-MA if a 3-Tesla magnet is used instead of the 0.3 Tesla used in this study. That means that a SWCNT-MA could be guided 1.5 cm below the skin at least. Today physicists can achieve 17-T fields with cryogenic equipment. However, the intensity of the field proved sufficient for modifying the aggregates orientation and position, and the cells appeared to be strongly attached to the SWCNT-MA.

We suggest that the nanoroughness of the SWCNT-MA is one of the factors responsible for stem cell aggregation. Nanoroughness can play a part on stem cell adhesion. There are few works in the literature about the subject [23].

Recently, a study showed that a similar system of gadolinium enriched SWCNT was successfully used as a magnetic retainer for cellular cardiomyoplasty [24]. In this study,* in vivo* epicardial cell injections were performed around a 1.3 T NdFeB ring magnet sutured onto the left ventricle of female juvenile pigs. However, an inflammatory response to the magnet was noted after 48 h [24]. Although improvements must be done, the technique is very promising.

## 4. Conclusions

The present study showed that modified SWCNT-MA, that is, SWCNT-MA consisting of SWCNT, molecular magnets, and peptide, could successfully anchor hASCs. The viable hASCs could be tilted and moved with an applied magnetic field that was 10 times lower than a commercial MRI scanner field. The technique described herein appears to be suitable for transporting stem cells throughout the body via the blood stream and may even be a viable method for guiding cells through narrow capillaries, being a promising noninvasive and nontoxic method. Further* in vivo* assays must be developed to prove this function and viability of the SWCNT-MA to guide cells through the body.

## Supplementary Material

A human adipose-derived stem cells aggregate is moved by an applied magnetic field. The field is applied by approaching a NdFeB magnet to the microscope table where lies the cell cultures.

## Figures and Tables

**Table 1:** Lineage-specific differentiation induction media.

Medium	Media	Serum	Supplementation
Control	DMEM, 5 mM sodium bicarbonate (Cinética Química Ltda), penicillin (100 units/mL, Sigma-Aldrich), streptomycin (0.1 mg/mL, Sigma-Aldrich), amphotericin B (0.25 *μ*g/mL, Sigma-Aldrich), gentamicin (60 mg/L, Schering-Plough).	10% FBS (Cripion Biotecnologia LTDA)	None

Adipogenic (AM)	DMEM, 5 mM sodium bicarbonate, penicillin (100 units/mL), streptomycin (0.1 mg/mL), amphotericin B (0.25 *μ*g/mL), gentamicin (60 mg/L).	10% FBS	0.5 mM isobutylmethylxanthine (Sigma-Aldrich), 200 *μ*M indomethacin (Sigma-Aldrich), 1 *μ*M dexamethasone (Aché), and 10 *μ*M insulin (Eli Lilly and Company).

Osteogenic (OM)	DMEM, 5 mM sodium bicarbonate, penicillin (100 units/mL), streptomycin (0.1 mg/mL), amphotericin B (0.25 *μ*g/mL), gentamicin (60 mg/L).	10% FBS	50 *μ*g/mL ascorbate-2-phosphate (Ecibra), 10 mM *β*-glycerophosphate (Sigma-Aldrich), and 0.1 *μ*M dexamethasone (Aché).

Chondrogenic (CM)	DMEM, 5 mM sodium bicarbonate, penicillin (100 units/mL), streptomycin (0.1 mg/mL), amphotericin B (0.25 *μ*g/mL), gentamicin (60 mg/L).	1% FBS	1 mM dexamethasone, 125 *μ*g/mL de BSA (PAA), 1 mM pyruvate (Sigma-Aldrich), 200 U/mL insulin, 3.25 *μ*g/mL transferrin (Wako), 0.01 *μ*g/mL TGF-*β*1 (Sigma-Aldrich), and 5 mg/mL ascorbate-2-phosphate.
